# Recombinant biosynthesis of functional human growth hormone and coagulation factor IX in transgenic soybean seeds

**DOI:** 10.1186/1753-6561-8-S4-P112

**Published:** 2014-10-01

**Authors:** Nicolau Brito da Cunha, André Murad, Giovanni Vianna, Elíbio Rech

**Affiliations:** 1 Embrapa Cenargen, Brasilia, Brazil

## Background

Plants constitute promising systems for the alternative production of valuable recombinant proteins. Recently, ELELYSO™, an enzymatic drug developed by the Israeli company Protalix Biotherapeutics to treat Type 1 Gaucher's disease, became the first plant-derived therapeutic product to reach marketable status [1]. This confirms the inherent potential of plant systems for the large-scale production of pharmaceuticals and industrial proteins with high quality and at competitive costs. Soybeans [*Glycine max *(L.) Merrill] provide a potential economically viable platform for the large-scale production of different therapeutic molecules. Plant seeds are specialized in the stable accumulation of proteins at high levels, representing an excellent source of abundant and cheap biomass. Additionally, the vegetative growth of plants can be significantly extended under a daily photoperiod of 23 h of light, inducing more than a tenfold increase in seed production when compared with plants cultivated under field conditions [2]. In this report we present the recombinant biosynthesis and the molecular and functional characterization of two important therapeutic proteins in transgenic soybean seeds: the 22 kDa human growth hormone (hGH) and the 56 kDa human coagulation factor IX (hFIX), a vitamin K-dependent serine-protease glycoprotein utilized to treat Type B Christmas disease, the second most frequent haemophilia variant [3,4].

## Methods

A biolistic process was used to introduce the plasmids pβcong3hGH (4,786 bp) and pbcong3FIX (5,406 bp), respectively carrying the *hgh *and *hFIX *coding sequences under the transcriptional control of the alpha prime (α') subunit of β-conglycinin tissue-specific promoter from *Glycine max *(L.) Merrill and the α-Coixin signal peptide from *Coix lacryma-jobi*, in soybean embryonic axes from mature seeds (cv. BR-16). Independent transgenic lines were analyzed by PCR and characterized by Southern blot. The transcription levels of the correspondent coding sequences were evaluated by RT-PCR of immature seeds and the accumulation of the proteins in the protein storage vacuoles (PSVs) of the seeds was detected by Western Blot and ultrastructural immunocytochemistry assays. Partial N-terminal sequencing of both proteins was determined by nano-Lc mass spectrometry assay. The biological activities of the molecules were evaluated by somatogenic activity bioassay (for the hGH), and by activated partial thromboplastin time assay (for the hFIX).

## Results

Expression levels of bioactive hGH and hFIX were, respectively, of up to 2.9% of total soluble seed protein content (corresponding to approximately 9 g kg^-1^) and up to 0.23% (0.8 g Kg^-1^). Immunocytochemistry assays indicated that both molecules were efficiently directed to protein storage vacuoles in seed cotyledonary cell. The recombinant hGH and hFIX protein sequences were confirmed by mass spectrometry characterization and showed no post-translational modifications on the spectra covered by the assays. The somatogenic activity bioassay demonstrated that the hGH expressed in soybean seeds is fully active. Protein extracts from transgenic seeds containing the hFIX showed a blood-clotting activity of up to 1.4% of normal plasma.

## Conclusions and perspectives

Soybean seeds seem to be promising vehicles for the stable accumulation of the recombinant hGH and hFIX, once it was possible to detect biologically active molecules in grains stored up to six years at room temperature.
